# Retrieval and Encoding Interference: Cross-Linguistic Evidence from Anaphor Processing

**DOI:** 10.3389/fpsyg.2017.00965

**Published:** 2017-06-09

**Authors:** Anna Laurinavichyute, Lena A. Jäger, Yulia Akinina, Jennifer Roß, Olga Dragoy

**Affiliations:** ^1^Neurolinguistics Laboratory, National Research University Higher School of EconomicsMoscow, Russia; ^2^Department of Linguistics, University of PotsdamPotsdam, Germany; ^3^Graduate School for the Humanities, University of GroningenGroningen, Netherlands; ^4^Speech Therapy Center ‘Uncle Tom's Cabin’Berlin, Germany; ^5^Department of Speech Pathology and Neurorehabilitation, Moscow Research Institute of PsychiatryMoscow, Russia

**Keywords:** encoding interference, retrieval interference, German, Russian, comprehension, reflexive processing, anaphor

## Abstract

The main goal of this paper was to disentangle encoding and retrieval interference effects in anaphor processing and thus to evaluate the hypothesis predicting that structurally inaccessible nouns (distractors) are not considered to be potential anaphor antecedents during language processing (Nicol and Swinney, 1989). Three self-paced reading experiments were conducted: one in German, comparing gender-unmarked reflexives and gender-marked pronouns, and two in Russian, comparing gender-marked and -unmarked reflexives. In the German experiment, no interference effects were found. In the first experiment in Russian, an unexpected reading times pattern emerged: in the condition where the distractor matched the gender of the reflexive's antecedent, reading of the gender-unmarked, but not the gender-marked reflexives was slowed down. The same reading times pattern was replicated in a second experiment in Russian where the order of the reflexive and the main verb was inverted. We conclude that the results of the two experiments in Russian are inconsistent with the retrieval interference account, but can be explained by encoding interference and additional semantic processing efforts associated with the processing of gender-marked reflexives. In sum, we found no evidence that would allow us to reject the syntax as an early filer account (Nicol and Swinney, 1989).

## 1. Introduction

In human language processing, working memory is crucial for linking together parts of syntactic dependencies. Therefore, to understand language processing it is important to understand mechanisms and limitations of the working memory system, especially those that lead to forgetting. Although previously attributed to decay (Brown, 1958), now forgetting is often believed to stem from similarity-based interference from other entities stored in memory (Nairne, 2002; Oberauer and Kliegl, 2006; Lewandowsky et al., 2008). Similarity-based interference may affect different working memory processes: writing (encoding) to memory, maintenance in memory, and retrieval.

### 1.1. Potential sources of similarity-based interference

Interference may arise during writing of an item to the working memory (encoding) if it shares some features with other items in memory. Such a model can be instantiated in different ways. One was proposed by Oberauer and Kliegl (2006): in their model, items in working memory are represented by sets of features that are activated together. If two items share the same feature (for example, two nouns share the same gender), they compete for it, and the competition may lead to so-called *feature overwriting*—loss of the feature in one of the sets. As a result, representation of an item that lost a feature gets less distinguishable, and the probability of the item's successful retrieval decreases. An alternative realization of encoding interference was proposed by Lewandowsky et al. (2008): when an item is first presented, its novelty is assessed in comparison to other items already stored in memory and their feature sets. If the item is judged to be novel, it is assigned greater encoding weight than if it is judged to be similar to the items in memory. The greater the encoding weight of an item, the easier it is to retrieve. Note, that although in both models interference arises during encoding of item's representation to the working memory, presence of interference affects retrieval of the item from memory.

Interference may also arise during the maintenance of an item in memory: if two or more items that share a certain feature are being stored in working memory, they may become less distinguishable from one another. The feature overwriting mechanism cited above can be thought of as maintenance interference depending on the time when the overwriting occurs. Consequently, maintenance interference is difficult to separate from encoding interference in practice, since we can only observe their effects at retrieval. Hence, in the following sections we do not distinguish between encoding and maintenance interference.

The third type of interference—retrieval interference—is assumed to arise during retrieval of an item from memory if other items share features relevant for retrieval with the target item. Among others, this type of interference is assumed in two memory retrieval models that have been applied to sentence processing: the Adaptive Control of Thought-Rational (ACT-R, see Lewis and Vasishth, 2005; Lewis et al., 2006; Anderson, 2014) and the working memory model by McElree (McElree, 2000; McElree et al., 2003; Martin and McElree, 2011). In the ACT-R model, each item is represented in memory as a bundle of features. To be retrieved, it must receive the highest activation among other items in memory. The activation of each item consists of its base-level activation (corresponding to the frequency and recency of its use), random noise, and spreading activation. Spreading activation is what an item receives during retrieval: to find a specific item in memory, each retrieval cue (such as a particular gender or case) propagates activation among all items which have a feature that matches the cue. The activation that each cue spreads is divided between all items that match this cue. According to ACT-R, this mechanism is the cause of similarity-based retrieval interference. The item whose features match all the retrieval cues receives the most spreading activation, which normally results in the highest boost of activation (modulo base-level activation and noise) and therefore reaches the activation threshold first (i.e., is retrieved from memory). Importantly, the activation of an item determines the speed of its retrieval: once an item reaches a certain activation threshold, it is retrieved, i.e., the stronger the boost in activation, the faster the retrieval. If there are competitor items that match some of the retrieval cues, they receive some spreading activation, As a result, less activation reaches the target, and the target is retrieved more slowly. Therefore, the ACT-R model predicts that retrieval interference leads to a processing slowdown.

In turn, McElree and colleagues (McElree, 2000; McElree et al., 2003; Martin and McElree, 2011) suggested that while items are retrieved from memory by means of retrieval cues, the retrieval speed remains constant irrespective of the number of competitors. But constant retrieval speed does not imply constant reading times: McElree proposes that reading times represent not only the retrieval speed, but also the probability of successful retrieval—if misretrieval occurs, parser initiates a reanalysis, which takes time. Consequently, according to McElree, reading times are not diagnostic of retrieval speed, only the speed-accuracy tradeoff paradigm allows us to tease apart retrieval probability and latency. In the studies presented in this paper, we will rely on the ACT-R framework and its predictions regarding the speed of retrieval (reflected in reading times) as an indicator of interference.

The types of interference listed above are not mutually exclusive: encoding/maintenance and retrieval interference can affect working memory independently, which is exactly what the Oberauer and Kliegl (2006) model assumes. In the psycholinguistic literature, there are very few experiments that pit the predictions of these types of interference against each other. Some exceptions—experimental results that clearly favor certain types of interference even if not rule out the others—will be reviewed below.

### 1.2. Interference effects in language processing

There are some similarity-based interference effects that can be explained only by interference arising during encoding and/or maintenance processes. The most notable example comes from the experiment of Gordon et al. (2001); replicated in Gordon et al. (2006), where participants were reading sentences such as (1):

(1)  a. It was the barber/John that___saw the lawyer/Bill in the parking lot.
       b. It was the barber/John that the lawyer/Bill saw___in the parking lot.

The authors reported that noun phrases differing in type (a common noun paired with a proper noun and vice versa) decrease reading times for object-extracted relative clauses (such as 1b)^1^ and increase question response accuracies. As retrieval occurs at the gap site, where no information about the noun type is provided, it cannot be retrieval interference that penalizes the processing of sentences with two nouns of the same type. On the contrary, encoding/maintenance interference easily accommodates these results: as the representation of similar items in working memory is degraded, retrieval of these items takes more time and is more error-prone.

In a different study, Gordon et al. (2002); see also Fedorenko et al., 2006) explored the influence of an increased memory load in a dual-task paradigm: the original sentences from Gordon et al. (2001)'s experiment with either both proper or both common nouns were preceded with triplets of proper (*Joel–Greg–Andy*) or common (*poet–voter–cartoonist*) nouns that participants had to memorize. As expected, the match between the type of nouns in memory and the ones in the sentence increased reading times and the number of errors in the answers to the comprehension questions. This effect was even stronger in the syntactically more complex object relative clauses. Again, only encoding interference can explain these results since there are no retrieval cues that could specifically trigger retrieval of only proper or common nouns and penalize the processing of sentences with similar noun types.

Retrieval interference effects, in turn, were demonstrated by Van Dyke and McElree (2006); see also Sekerina et al. (2016) in a memory-load paradigm similar to Gordon et al.'s (2002) experiment (2):

(2)  a. table–sink–truck/∅
           It was the boat that the guy who lived by the sea *sailed* in 2 sunny days.
       b. table–sink–truck/∅
           It was the boat that the guy who lived by the sea *fixed* in 2 sunny days.

While Gordon et al. (2002) manipulated the similarity between the memory load and the retrieval target, Van Dyke and McElree (2006) manipulated the match between the memory load and the retrieval cues provided by the semantics of the verb. As a result, reading times at the verb increased in condition 2b as compared to 2a, but only when a memory set was present. The authors interpret these findings as evidence for interference during cue-based retrieval: semantic retrieval cues provided by the verb *sailed* can uniquely identify the to-be-retrieved item in memory (boat), while the cues provided by the verb *fixed* are compatible with all the items held in memory (table, sink, truck, and boat), which causes interference during retrieval and, therefore, a processing slowdown.

In another study, Van Dyke (2007); see also Van Dyke and Lewis (2003) explored both syntactic and semantic interference arising within one sentence. Participants were presented with items such as (3):

(3)  The worker was surprised that the resident…
       a. who was living near the dangerous warehouse
       b. who was living near the dangerous neighbor
       c. who said that the warehouse was dangerous
       d. who said that the neighbor was dangerous
       …was complaining about the investigation.

The authors reasoned that to retrieve the subject while processing a verb, syntactic as well as semantic retrieval cues may be used, and indeed, a slowdown was found both in conditions with syntactic (3c and 3d) as well as semantic (3b and 3d) distractors. Note, that these results are compatible with the encoding interference account: during encoding and maintenance both semantically and syntactically similar nouns would be predicted to lose features they share, and hence would be more difficult to retrieve. Basically, both encoding and retrieval interference accounts predict identical results in this setup. The same criticism applies to Van Dyke and McElree's (2011) study with similar experimental conditions as well as to studies by Martin and McElree (2009, 2011).

Therefore, although many studies are conducted with the retrieval interference framework in mind, few experiments clearly demonstrate the effects of retrieval interference that cannot be explained by interference during memory encoding/maintenance. Also, it should be noted that the only unambiguous evidence for retrieval interference comes from experiments manipulating semantic cues (Van Dyke and McElree, 2006; Van Dyke, 2007). There is, however, a common potential limitation in the studies discussed so far: they explore interference in subject-verb and filler-gap dependencies, where the second part of the dependency is predictable as soon as the first is encountered (e.g., encountering a filler posits existence of a gap later in the sentence); therefore, subjects and fillers might be maintained in focal attention (McElree, 2006), and not retrieved at encountering the verb or the gap. A more convincing demonstration of retrieval interference would come from a dependency where the first element does not posit the existence of the second, such as a retrieval of a pronoun's or a reflexive's antecedent. Indeed, many studies are investigating interference in anaphor resolution. We will discuss these studies next.

### 1.3. Interference effects in anaphor processing

In syntax, the Binding Theory (Chomsky, 1981) identifies strict syntactic constraints defining the set of grammatical antecedents for pronouns and reflexives. The question whether these constraints are considered from the early stage in online processing (Nicol and Swinney, 1989) or applied as a later filter (Badecker and Straub, 2002) has been studied extensively. Researchers tested whether distractors that are not licit antecedents of pronouns and reflexives affect anaphor resolution.

In pronouns, clear interference effects were found in some studies, as in Badecker and Straub (2002) (4):

(4)  a. John thought that Bill owed him another chance to solve the problem.
       b. John thought that Beth owed him another chance to solve the problem.

In condition 4a where both the antecedent and the structurally inaccessible distractor match in gender, reading times after the pronoun *him* were elevated in comparison to condition 4b. These results are interpreted as demonstrating interference from the distractor, and the authors conclude that grammatical constraints do not rule out grammatically illicit attachment sites at an early stage of processing. This conclusion was supported by a number of other studies (Kennison, 2003; Clackson et al., 2011; Runner and Head, 2014). However, note that several experiments failed to observe interference effects in pronouns (Chow et al., 2014; Patterson et al., 2014; Cunnings et al., 2015).

In reflexive binding a contradictory pattern of results is emerging: many studies found interference effects, which is inconsistent with the syntax as early filter account (Sturt, 2003), but at least as many other studies did not. For example, Badecker and Straub (2002) reported a slowdown two words downstream the reflexive when distractor matched the gender of the reflexive's antecedent (from now on, *interference* condition), as in (5):

(5)  a. Jane thought that Bill owed himself another opportunity to solve the problem.
       b. John thought that Bill owed himself another opportunity to solve the problem.

Similar results were observed in several other studies (Nicol and Swinney, 2003; Chen et al., 2012; Clackson and Heyer, 2014; Jäger et al., 2015a, Experiments 1 and 2; Jäger et al., 2015b, Experiment 2 in grammatical conditions, Experiment 1 in ungrammatical conditions; and Patil et al., 2016). In addition, several studies reported a speed-up in the interference condition (Sturt, 2003, Experiment 1; Cunnings and Felser, 2013, Experiment 2; Cunnings and Sturt, 2014; Baumann and Yoshida, 2015; Jäger et al., 2015a, Experiment 3). Overall, in a meta-analysis (Jäger et al., 2017) no evidence was found for interference in experiments on reflexives with materials such as 5a and 5b. We will discuss the slowdown vs. speed-up interference effects in more detail in Section 3.5.

Interference effects were also found in a visual-world eye-tracking paradigm: Runner and Head (2014); see also Clackson and Heyer (2014) demonstrated that distractors matching the gender of the antecedent attracted participants' attention from the onset of the reflexive more than gender-mismatching distractors, which means that participants at least sometimes attempted to bind the reflexive to the distractor. The same effects were also found in children (Clackson et al., 2011). It is not straightforward to decide whether this result patterns with a slowdown or a speed-up in reading times, but it clearly demonstrates the presence of interference effects.

However, as mentioned earlier, many experiments failed to observe any interference effects (Nicol and Swinney, 1989; Clifton et al., 1999; Badecker and Straub, 2002, Experiments 5, 6; Sturt, 2003, Experiment 2; Xiang et al., 2009; Clackson et al., 2011; King et al., 2012; Dillon et al., 2013; Kush and Phillips, 2014; Parker and Phillips, 2016). We will return to this point and discuss possible reasons for the lack of interference effects in reflexive processing later in this paper. For a more in-depth literature review of interference effects in reflexives, refer to Jäger et al. (2017).

Most studies that targeted similarity-based interference in reflexives did not explicitly aim to test which type of interference affects reflexive processing (one exception is Jäger et al., 2015a), but rather assumed that interference arises during retrieval, when the parser is processing the reflexive and triggers the search for its antecedent. Since in most languages in which the studies were conducted, reflexives are gender- and number-marked, the reflexive's gender and number are likely to be used as retrieval cues, and all the items in memory with features that match those cues would compete for retrieval. Thus, whenever interference effects were found, they were attributed to this competition for retrieval and seen as evidence against syntax as an early filter account (Nicol and Swinney, 1989). However, Dillon et al. (2013) suggested that it might be not retrieval, but rather encoding interference that influenced the processing of reflexives. Within the encoding interference framework, if two (or more) words with the same gender and number marking are encoded to the working memory, the representation of these words would be degraded, and retrieval of those words would take more time and fail more often. If this hypothesis turns out to be true, interference effects in the literature cannot be interpreted as unambiguous evidence for retrieval interference and hence as evidence against syntax as an early filter account.

Jäger et al. (2015a) tested the encoding interference account and its predictions directly: in German, the reflexive *sich* is not gender-marked; as a result, gender cannot be used as a retrieval cue. Consequently, retrieval interference is not expected to influence the processing of sentences with gender match between the antecedent and distractor in German. In contrast, encoding interference is expected to occur any time two similar items are written to working memory, and would manifest itself in longer retrieval times and more retrieval errors. In two experiments with relatively large number of participants, Jäger et al. (2015a) found no slowdown at or after the reflexive region and concluded that there is no evidence for encoding interference affecting online reflexive processing. However, some concerns were raised, mainly that the null result does not prove the absence of an effect. In Experiment 3 on Swedish possessives, a more direct evidence in favor of retrieval interference was found: fewer first-pass regressions were observed in the interference condition when possessives were gender-marked in contrast to the gender-unmarked. However, as possessives might be processed differently than reflexives, the conclusions one might draw from this result are still limited.

This brings us directly to the main point of the present paper: to find out whether it is encoding or retrieval interference that affects anaphor processing. The first of the three presented experiments contrasts reflexive and pronoun processing in German: an interference effect in pronouns and an absence of the effect in reflexives in the same sample would provide more convincing evidence against encoding interference.

## 2. Experiment 1: german reflexives and pronouns

As mentioned above, reflexives do not bear any gender marking in German; therefore, the gender feature cannot be used for retrieval, and no retrieval interference is expected if the antecedent and the distractor share the same gender. In contrast, German pronouns are gender-marked, hence gender might be used for the retrieval of the pronoun's antecedent. If we observe interference effects in pronouns but not reflexives, one can conclude that the source of interference is the retrieval process rather than processes happening during encoding or maintenance. On the other hand, if we find interference effects both in pronouns and reflexives, retrieval interference is not able to account for that pattern and we can conclude that the interference is caused by processes during memory encoding or maintenance.

### 2.1. Materials and methods

We designed 42 sets of experimental items, manipulating interference (match or mismatch in gender between the antecedent and the distractor) and dependency type (reflexive, pronoun, or a noun phrase that does not trigger retrieval). This resulted in a 2×3 design, see Example (6). Sentences were constructed such that the reflexive/pronoun preceded the main verb in order to avoid reactivation of the antecedent before processing the anaphor. Both the antecedent and the distractor were subjects of their respective clauses and had nominative case marking in order to increase the chance to observe an effect (there is evidence suggesting that distractors in subject position induce stronger interference, see Jäger et al., 2017). The experimental items consisted of three clauses: the main clause served as preface, while the subordinate clauses contained the actual experimental manipulation. We opted for this structure since only in a subordinate clause does German syntax allow the reflexive/pronoun to precede the main verb. The subordinate clause contained a subject (the antecedent of the reflexive) modified by a dative relative clause with the distractor in subject position, matching, or mismatching the reflexive's antecedent in gender. Note, that while for reflexives the antecedent is the subject of the second clause and the distractor is the subject of the dative relative clause, it is the reverse for the pronouns: the subject of the second clause is the distractor and the subject of the dative relative clause is the antecedent. We will discuss the materials with focus on the reflexive condition, but keep in mind that the order of target and distractor is reversed in the pronoun condition. The dative relative clause was followed by a direct object that triggered the retrieval in the pronoun/reflexive conditions. In the control condition, this direct object was an animate noun phrase in neuter gender. Thus, no retrieval is triggered at the critical word, and therefore no difference between the interference and no interference conditions is expected. The spillover region was constant across conditions and contained a prepositional phrase and a verb. The experimental materials were additionally balanced by gender of the antecedent (21 items with a masculine and 21 with a feminine antecedent).

All materials, results and analysis files for all the experiments reported in this paper can be downloaded from Open Science framework (https://osf.io/xfthm/).

  (6)  a.  **Interference**
              Das Journal schreibt, dass der       Βürokrat,
              The journal writes      that the_masc_ bureaucrat_i_
              dem           der       Schriftsteller geraten hat
              the_Dat, masc_ the_masc_ writer_j_         advised
              umzudenken, **sich/ihn/das Mitglied**        in dem
              to reconsider **self_i_/him_j_/the_neu_ member** in the
              gigantischen Einkaufszentrum blamiert hat.
              giant            mall                    embarrassed has.
         b.  **No interference**
              Das Journal schreibt, dass der       Βürokrat,
              The journal writes      that the_masc_ bureaucrat_i_
              dem           die       Schriftstellerin geraten hat
              the_Dat, masc_ the_fem_ writer_j_             advised
              umzudenken, **sich/sie/das Mitglied**        in dem
              to reconsider **self_i_/her_j_/the_neu_ member** in the
              gigantischen Einkaufszentrum blamiert hat.
              giant             mall                   embarrassed has.
              “The journal writes that the bureaucrat, whom
              the the male/female writer advised to rethink,
              embarrassed himself/(him/her)/the member in the
              giant mall.”

Each sentence was followed by a yes/no comprehension question (see Example 7). Half of the questions asked about the antecedent, and the other half about the distractor. The questions were balanced with regard to the number of yes/no answers. They were designed in such a way as to not repeat the lexical material of the corresponding sentence and required deep semantic processing of the sentence.

 (7)  Blieb dem     Βürokraten     eine Blamage           erspart?
        Was   the_Dat_  bureaucrat_Dat_ an    embarrassment spared?
        Was the bureaucrat spared   the   embarrassment?

Experimental items were mixed with 83 filler sentences.

Participants completed a moving-window self-paced reading experiment programmed in Linger (Rohde, 2005). The order of presentation was pseudorandomized such that each experimental item was followed by at least one filler; each session started with five practice trials to help participants get used to the task.

### 2.2. Participants

One-hundred and eleven participants were tested at the University of Potsdam in exchange for course credit or payment of five Euros. All participants were neurologically healthy native speakers of German, mostly students of the University of Potsdam. Their demographic data were not recorded.

### 2.3. Analysis

Nicenboim et al. (2015) provide persuasive evidence that participants who do not complete syntactic dependencies and resort to guessing the answer to the comprehension questions process linguistic input qualitatively different from participants who answer questions correctly: individuals who fail to build a correct representation of the sentence read the critical retrieval region faster. Therefore, it is undesirable to conflate the data from these different categories of participants in one analysis: the slowdown in reading times of accurate participants might be concealed by a speedup in reading times of participants who do not parse the syntactic structure correctly. To avoid this, we included mean accuracy in answering the comprehension questions to experimental items as a predictor in the models of reading times. Mean participant accuracy is a reasonable approximation of the probability with which any given trial would be processed successfully by certain participant. We decided against trial accuracy because of the implicit assumption that every trial which resulted in a correct response was processed successfully. This is not necessarily true: a participant might fail in processing most of the trials but still provide correct responses for half of them due to chance. Mean subject accuracy better accounts for such cases at the expense of trial level variation.

We fit linear mixed-effects models using R (R Core Team, 2016) to the reading times from four regions: (*a*) the relative clause participle (*umzudenken*); (*b*) the critical region containing reflexive, pronoun, or NP (*sich/(ihn/sie)/das Mitglied*); (*c*) the preposition and article after the critical region (*in dem*); and (*d*) the adjective (*gigantischen*)^2^.

For analysis, reading times were log-transformed. Whenever, the residuals were not normally distributed, we checked whether deletion of problematic data points changed the results using the package “influence.ME” (Nieuwenhuis et al., 2012). In no case did exclusion of problematic data points change the results. For linear mixed-effects models, the “lme4” package version 1.1-8 (Bates et al., 2015) was used. Sum contrast coding was used to test the main effects and interactions. In addition, pairwise comparisons were modeled by applying sum contrasts nested within each level of dependency type factor whenever the interaction was significant.

For the analysis of response accuracies, linear mixed-effects models with a logistic link function were used. The model of question response accuracy included main effects of dependency type and interference as well as by-subject and by-item random intercepts and slopes for the main effects, but not for the interaction due to non-convergence of the full model.

The reading times models included main effects of interference, dependency type, and mean participant accuracy (centered and scaled, i.e., *z*-scores), the three-way interaction between them, as well as two-way interactions between dependency type and interference, and accuracy and interference. The random part of the models included random intercepts for subjects and items as well as by-item random slopes for all main effects, and by-subject random slopes for the main effects of match and dependency type. As mean accuracy is a between- rather than within-subjects predictor, it was not included into by-subject random slope structure. Interactions between main effects were also not included in the random effects structure of the model due to convergence problems.

### 2.4. Results

#### 2.4.1. Accuracy

The mean accuracy rates across conditions and the corresponding standard errors are presented in the Table 1.

**Table 1:** Experiment 1: Mean accuracies and standard errors by conditions.

	**Noun phrase**	**Pronoun**	**Reflexive**
Interference	0.63 (0.018)	0.56 (0.019)	0.61 (0.018)
No interference	0.67 (0.018)	0.70 (0.018)	0.69 (0.018)

Mean accuracies by participant ranged from 0.40 to 0.90, with a mean of 0.64. Fifty-three out of one-hundred and eleven participants had mean accuracies below chance level (defined as the highest number of mistakes a participant could make such that exact binomial test would still result in a *p*-value of 0.05 or lower, indicating that the number of correct responses was above chance; 14 mistakes in this experiment).

Statistical analysis revealed a main effect of interference: accuracy was lower in the condition where the antecedent and the distractor shared the same gender ($\hat{\beta }=-0.46$, *SE* = 0.11, *z* = 4.07, *p* < 0.001). There was a significant interaction between the effect of interference and the dependency type ($\hat{\beta }=$ 0.25, *SE* = 0.10, *z* = 2.44, *p* = 0.02). The model with pairwise comparisons revealed that in the conditions with reflexives and pronouns as compared to nouns, accuracy was lower when the antecedent and the distractor shared the same gender ($\hat{\beta }$ = −0.45, *SE* = 0.14, *z* = −3.28, *p* < 0.01 for reflexives; $\hat{\beta }=-0.81$, *SE* = 0.26, *z* = −3.05, *p* < 0.01 for pronouns), but the effect was not present in the control condition with nouns.

#### 2.4.2. Reading times

**Figure 1:**
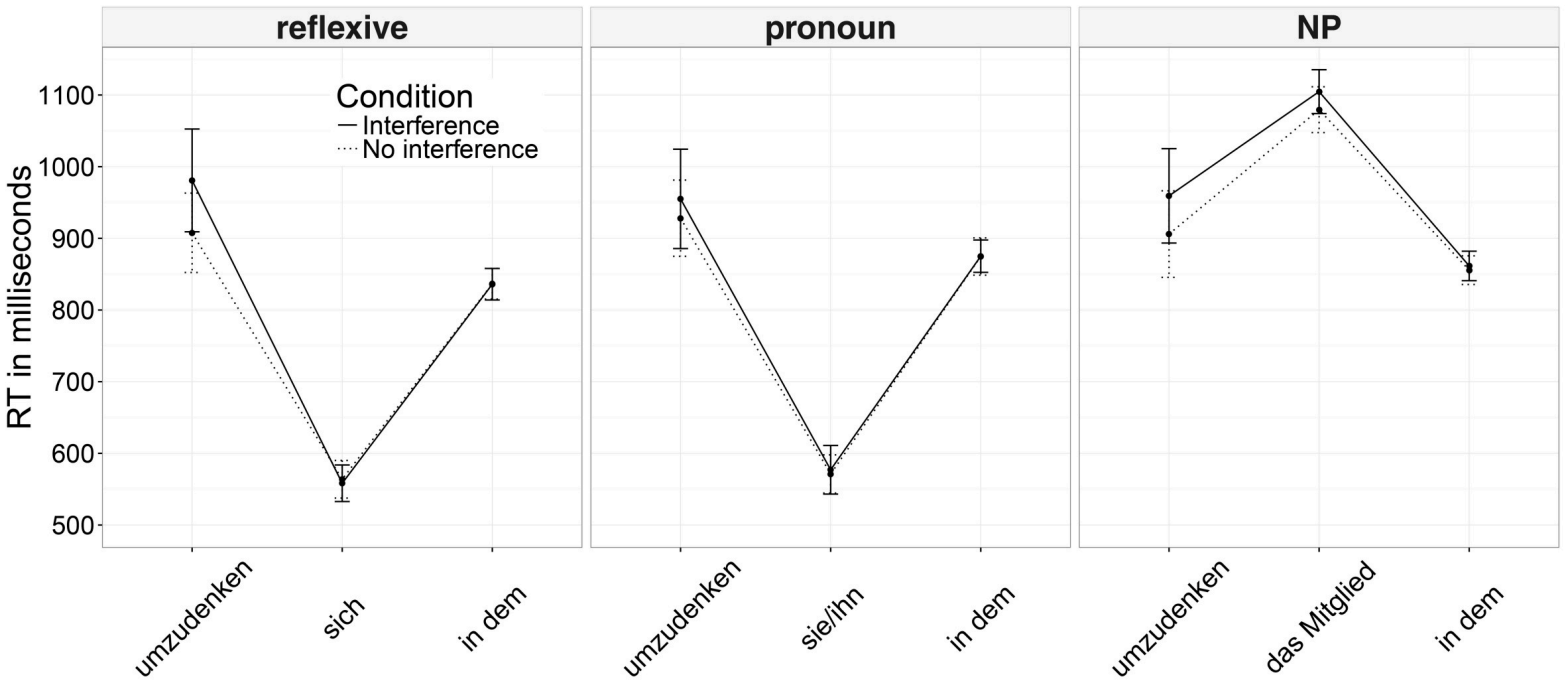
Mean reading times across conditions and their confidence intervals (Experiment 1).

Mean reading times and their respective confidence intervals for the analyzed regions across conditions are presented in Figure 1.

**Figure 2:**
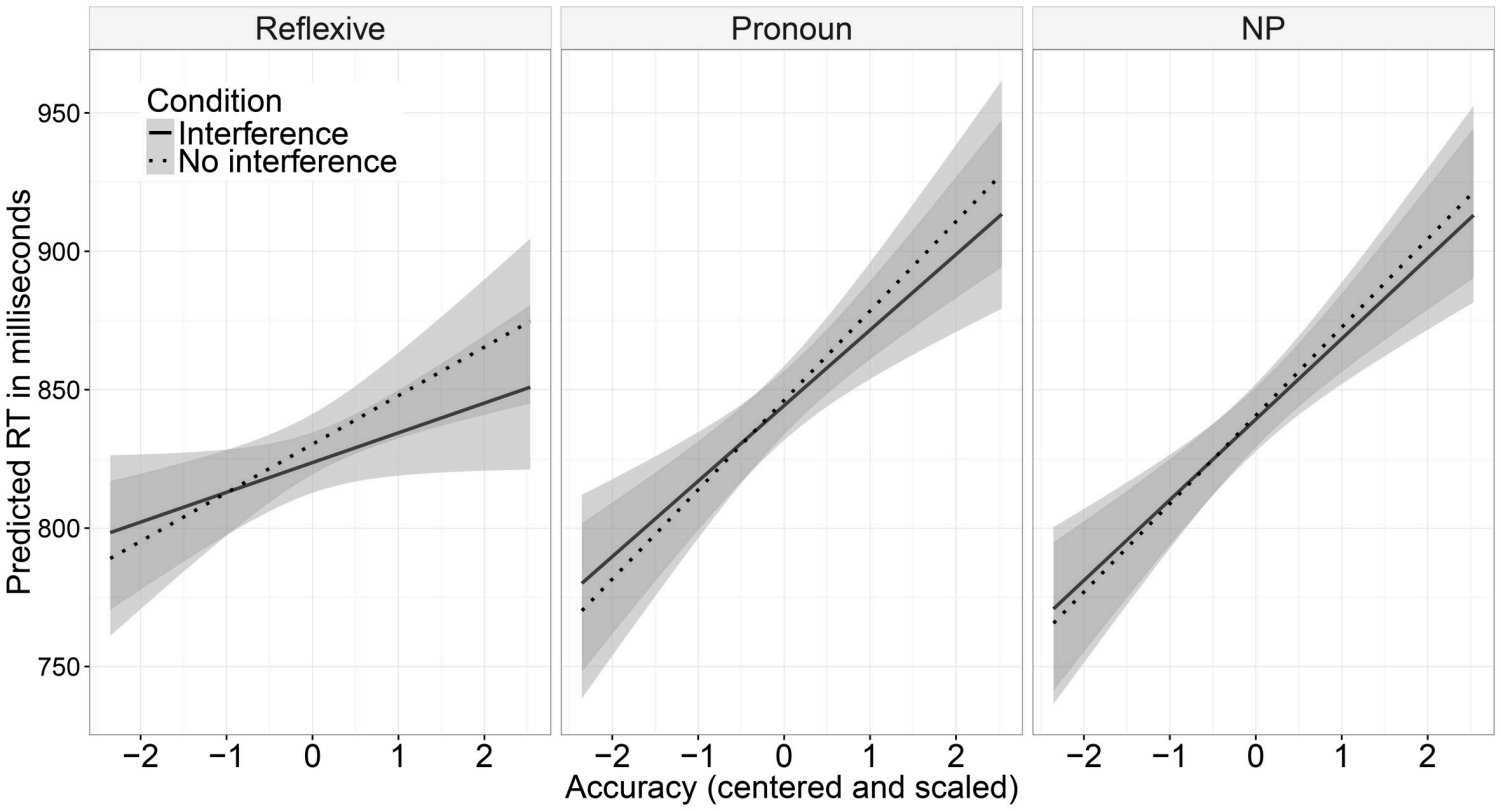
Modeled reading times (and respective standard errors) at the spillover after critical region (Experiment 1).

In the pre-critical region (the verb *umzudenken* in Example 6), a significant main effect of participants' mean accuracy was found (see Table 2): more accurate participants read the region more slowly. There was also a significant three-way interaction between interference, dependency type, and accuracy, but since the conditions were identical for both dependency types at that region, we discard this result as a Type I error. In the critical region, dependency type significantly affected reading times: both reflexives and pronouns were read faster than nouns. There was also a significant main effect of accuracy: the region was read more slowly by the more accurate participants. For the analysis of reading times in the post-critical region by-item random slopes for the main effects of dependency type and accuracy were removed due to non-convergence of the model. We opted for eliminating by-item random slopes since by-item variance is usually smaller than by-subject. In this region, again, dependency type significantly affected reading times: the region was read faster in conditions where the direct object was a reflexive in comparison to a noun. There was also a three-way interaction between dependency type, interference, and accuracy (see Figure 2). Nested contrasts demonstrated that the interaction was driven by a two-way interaction between accuracy and dependency type: mean accuracy had less influence on the speed of reading the post-critical region after reflexives than after nouns ($\hat{\beta }=-0.012$, *SE* = 0.004, *t* = −3). No other comparisons were significant in any region.

**Table 2:** Experiment 1: Main effects of interference, dependency type, accuracy, and their interaction on log-transformed RTs by regions.

	**Pre-critical ***umzudenken*****		**Critical ***sich/{ihn/sie}/das Mitglied*****		**Post-critical 1 ***in dem*****		**Post-critical 2 ***gigantischen*****	
	**$\hat{\beta }$(*SE*)**	***t***	**$\hat{\beta }$(*SE*)**	***t***	**$\hat{\beta }$(SE)**	***t***	**$\hat{\beta }$(*SE*)**	***t***
Interference	0.011(7)	1.46	0.002(5)	0.32	−0.002(3)	−0.7	0.003(4)	0.71
Reflexive vs. NP	0.004(12)	0.36	−0.234(8)	−29.45	−0.010(4)	−2.4	−0.001(54)	−0.02
Pronoun vs. NP	0.011(12)	0.92	−0.223(9)	−25.16	0.006(4)	1.5	−0.001(5)	−0.22
Accuracy	0.136(47)	2.90	0.058(22)	2.53	0.029(18)	1.6	0.024(25)	0.97
Interf.×Refl.	0.008(17)	0.77	−0.003(7)	−0.38	−0.002(4)	−0.5	0.001(5)	0.19
Interf.×Pron.	−0.014(10)	−1.37	−0.005(7)	−0.74	0.001(3)	0.2	−0.007(5)	−1.26
Interf.×Acc.	0.005(7)	0.66	−0.004(5)	−0.73	−0.003(3)	−0.9	0.005(4)	1.41
Interf.×Refl.×Acc.	−0.033(16)	−2.06	0.002(10)	0.16	−0.013(6)	−2.2	0.002(8)	0.20
Interf.×Pron.×Acc.	0.027(15)	1.74	−0.006(10)	−0.61	0.006(6)	0.9	−0.003(8)	−0.39

### 2.5. Discussion

The comparison of interference effects in reflexives and pronouns revealed that question response accuracy was lower in the conditions with reflexives and pronouns when the antecedent and the distractor shared the same gender. The effect was not present in the control condition. This pattern can be explained by encoding interference, but is inconsistent with retrieval interference: when the distractor shares the gender of the antecedent, accuracy is lower independently of the anaphor type: interference is present both in gender-unmarked reflexives and in gender-marked pronouns. No difference in accuracy in the control condition with nouns is consistent with the notion that interference manipulation affects only those sentences where retrieval of the antecedent should happen. This pattern replicates the findings for German reflexives reported by Jäger et al. (2015a) in Experiments 1 and 2. However, question response results should be interpreted with caution since we were primarily testing the predictions of the interference accounts with respect to the reading times, and comprehension question accuracies might reflect processes different from those of online sentence comprehension.

It is also unclear, why the overall question response accuracy was so low. It might be the case that the double nested syntactic structure was too challenging for our participants. Another factor that might have affected participants' performance was the nature of comprehension questions (see Example 7): answering the question correctly required making inferences about the situation described in the experimental sentence, and not just remembering the propositions. To our knowledge, comprehension questions in most experiments are easier to answer and probe either the superficial understanding of the sentence (“Was anyone embarrassed?”) or the dependency resolution (“Who was embarrassed?”). It might be possible that the combination of the double nested syntactic structure together with the demanding comprehension questions was too difficult for many participants.

An interesting point that does not directly relate to the main purpose of the study is that for the pre-critical and critical regions we found that participants' mean accuracy and reading times are correlated: participants who resolve syntactic dependencies correctly read more slowly (see also Ferreira et al., 2002). This replicates and extends the findings of Nicenboim et al. (2015) that participants who do not answer comprehension questions correctly tend to rush through the retrieval site. In our case, the effect is present not only at the retrieval site, but also at the pre-critical region. It is probable that less accurate participants might read the whole sentence more quickly. This might be explained by the limitations of working memory resources: those participants with lower WM capacity try not to lose the unresolved dependencies they have to keep track of, and speed up in order to resolve the dependencies and lift the burden as quickly as possible. However, since we did not measure participants' working memory, this must remain a speculation.

Unfortunately, we found no main effect or interactions involving the interference manipulation in reading times, and thus no evidence in favor of either encoding or retrieval interference. If anything, this suggests that there are no interference effects in the processing of anaphor dependencies, but one must be cautious interpreting the absence of the effect in favor of the null hypothesis. In addition, comparing reflexives with pronouns is potentially problematic. Interestingly, we found that the post-critical region was read faster when the critical region contained a reflexive in comparison to a noun (and even faster by more accurate participants). No such speedup was present in the post-critical region after a pronoun, although both reflexives and pronouns were read faster than nouns in the critical region. The fact that this speedup was independent of the interference manipulation suggests that it might reflect syntactic processing differences between reflexives and pronouns, whose interpretation is subject to different syntactic constraints. A better experimental design would allow us to compare gender-marked with gender-unmarked reflexives, which is not possible either in English or in German. Luegi et al. (2016) contrasted gender-marked and gender-unmarked reflexives in Portuguese, but did not find any difference in online processing. One of the possible reasons could be that in European Portuguese, the gender-marked reflexives are split constructions: first, a reader encounters an unmarked reflexive (*se*), then a verb, and only after the verb comes the gender-marked part of the reflexive (*a si mesmo/mesma*). In such configuration, retrieval is triggered at encountering the first, gender-unmarked, part of the reflexive. A better experimental design is possible in Russian, which allows us to test different interference accounts' predictions within one language.

## 3. Experiment 2A: russian reflexives, reflexive precedes the verb

Russian has two types of reflexives with the same syntactic distribution and with binding rules generally close to those of English and German (analogous in all aspects relevant for our research question; for more detail on Russian reflexive binding, see Rappaport, 1986): gender-unmarked *sebja* (similar to German *sich*) and gender-marked *samu/samogo sebja* (similar to English *herself/himself*). This provides us with an opportunity to pit retrieval and encoding interference predictions directly against each other: the encoding interference account would predict the slowdown in the conditions where the distractor shares the gender of the antecedent, irrespective of the reflexive type. The retrieval interference account, in turn, would predict an interaction between the reflexive type and the presence/absence of interference: only in the gender-marked reflexives would gender be used as a retrieval cue; and hence we should expect an interference effect only in the gender-marked reflexives, but not in the gender-unmarked reflexives.

### 3.1. Materials and methods

We designed 32 sets of experimental items, manipulating in a 2×2 design the interference and type of reflexive (gender-unmarked *sebja* vs. gender-marked *samogo*/*samu sebja*). Experimental items consisted of a main clause and an embedded relative clause (see Example 8). The main clause subject, the reflexive's antecedent, was followed by an object-extracted relative clause containing the distractor noun (matching or mismatching the main clause subject in gender) in subject position. The relative clause was followed by the reflexive (gender-marked or gender-unmarked), an adverb, and the main clause verb. All the verbs were in present tense in order to avoid the gender marking on the verbal past in Russian. Additionally, in the relative clause all nouns except for the distractor had neutral gender.

  (8)  a.  **Interference**
              Аферистка_i_, которую **торговка**         нанимает
              Swindler_fem_   whom      **merchant_fem_**    hires
              для ограбления, **себя_i_/саму себя_i_**
              for   robbery,        **self**_**acc**(ø)_/**herself_acc(fem)_**
              серьёзно     переоценивает в  способности к
              significantly   overestimates     in  ability              to
              обману.
              do trickery.
         b.  **No interference**
              Аферистка_i_, которую **торговец**           нанимает
              Swindler_fem_   whom      **merchant_masc_**    hires
              для ограбления, **себя_i_/саму себя_i_**
              for   robbery,        **self**_**acc**(ø)_/**herself_acc(fem)_**
              серьёзно     переоценивает в  способности к
              significantly   overestimates     in  ability              to
              обману.
              do trickery.
              “The swindler_fem_, whom a merchant_masc/fem_ hires
              for  a   robbery,  significantly   overestimates   her
              own_ø/fem_ trickery skills.”

Within an experimental item, the antecedent and both the matching and mismatching distractors had the same length (counted in number of syllables), and their lemma frequency never exceeded 100 tokens per million (Lyashevskaya and Sharov, 2009). Experimental materials were additionally balanced by gender of the antecedent (16 masculine, 16 feminine) and by noun type (16 experimental items had proper nouns, and 16 had common nouns). We employed proper nouns because distractors had to differ in gender but have the same word length within each item, and Russian has a very limited number of such common noun pairs.

Within an experimental item, the difference in frequency between matching and mismatching distractors did not exceed 50 tokens per million in common nouns and 10 tokens per million in proper nouns. The difference in frequency between the feminine and masculine antecedents across items was not significant, and neither was the difference between matching and mismatching distractors across items^3^.

The structure of 32 filler sentences superficially resembled the one of the experimental items in order to hide the experimental manipulation effectively. Each filler sentence consisted of a main and an embedded relative clause, but in contrast to the experimental items, the relative clause was subject-extracted. This discouraged participants from developing a strategy to process every sentence as containing an object relative clause, and encouraged deep structure processing. In fillers, the nouns in the main and the relative clauses had the same gender in half of the filler sentences. The fillers were additionally balanced by gender of the first noun (16 feminine, 16 masculine) and by noun type (16 fillers had proper, and another 16 had common nouns). Instead of a reflexive, a verb with a reflexive postfix (-*sja*, which does not necessarily convey reflexive meaning in Russian) was used. An example of a filler sentence is given below in (9):

(9)  Студент,       который зазвал приятеля   на
       Student_masc_, who         invited friend_masc_  to
       вечеринку, основательно закупается продуктами.
       party,          a lot                 buys_sja_         of food.
       “A student who invited his friend to a party buys a lot of food.”

Each sentence was followed by a wh- comprehension question with two answer options to choose from (see an example comprehension question for the experimental item in 10). In experimental items, 11 questions probed for the antecedent, 11 for the distractor, and 10 superficial questions probed for the adjuncts. To distract participants from the reflexive-antecedent dependency, in filler sentences, 20 questions probed for the adjuncts, six probed for the subject of the main clause, and the remaining six probed for the object of the relative clause. Questions were counterbalanced within each experimental list. In the questions, neither lexical reflexives nor the lexical material from the experimental items were used to discourage superficial processing.

(10)  Κто высоко оценивает свои способности?
        Who highly   thinks of     own  abilities?
        “Who thinks highly of his/her own abilities?”

Each participant was assigned to one of four experimental lists arranged in a Latin square design. Each list consisted of 32 experimental items (each participant saw only one version of each item) and 32 fillers (the same across the lists). The order of experimental items and fillers was pseudo-randomized and controlled for the noun type (proper/common, maximum two of the same type in a row), question type (no more than three questions of the same type in a row) and for sentence type (experimental item/filler, no more than two of the same type in a row). In the beginning of each experimental session, the participant saw four training items. Position of correct answers on the screen had a different randomization for each trial and participant.

### 3.2. Participants

One-hundred and nine volunteers completed a moving-window self-paced reading experiment programmed in Linger (Rohde, 2005). All participants were neurologically healthy native speakers of Russian, tested either at the Higher School of Economics (Moscow) or at the “Russian Reporter” Summer School. Mean age of participants was 21 (range 16–65), 17 out of 109 participants were male, 2 individuals reported to be left-handed. The study was approved by the Committee on Interuniversity Surveys and Ethical Assess of Empirical Research of the National Research University Higher School of Economics.

### 3.3. Analysis

The analysis was equivalent to the one described for the experiment on German (Section 2.3). The comprehension questions' responses were analyzed using a generalized linear mixed model with a logistic lin kfunction. The model included main factors of reflexive type and interference as well as interaction between them. The random effects structure included by-subject and by-item random intercepts and slopes for the main effects and their interaction.

As in Experiment 1, for reading time analyses, we computed participants' mean accuracy scores in answering the antecedent- and distractor-probing questions and used these scores as predictors. The linear models included main effects of reflexive type, interference, and accuracy, as well as the three-way interaction between these, the two-way interactions between reflexive type and interference, and accuracy and interference. The random effects structure included by-participant and by-item random intercepts and slopes for all the effects included in the model. By-participant random slopes did not include accuracy, as accuracy is a between-subjects predictor. For all linear models, correlations between random effects were not estimated.

We analyzed reading times data from the following four regions: (*a*) the region preceding the reflexive (*for a robbery*); (*b*) the reflexive (*sebja/samu sebja, self/herself*); (*c*) the spillover after the reflexive (*significantly*); and (*d*) the main clause verb (*overestimates*). Note, that the reflexives *sebja* and *samogo/samu sebja* were presented and analyzed as one region. Consequently, we expected to find a trivial main effect of reflexive type in reading times: the gender-marked reflexive should take more time to be read simply because the region is longer.

### 3.4. Results

#### 3.4.1. Accuracy

The mean accuracy rates across conditions and the corresponding standard errors are presented in the Table 3.

**Table 3:** Experiment 2A: Mean accuracies and standard errors across conditions.

	**Gender-marked**	**Gender-unmarked**
Interference	0.81 (0.014)	0.81 (0.014)
No interference	0.88 (0.012)	0.87 (0.012)

Mean partipiants' accuracies in answering the antecedent- and distractor-probing questions ranged from 0.45 to 1.00, with a mean of 0.79. Thirty-three subjects out of one-hundred and nine scored on average below chance (made more than six mistakes).

Statistical analysis revealed a main effect of interference: accuracy was lower in the conditions where the antecedent and the distractor shared the same gender ($\hat{\beta }=-0.31$, *SE* = 0.05, *z* = −5.86, *p* < 0.001). The effect of reflexive type and the interaction were not significant.

#### 3.4.2. Reading times

**Figure 3:**
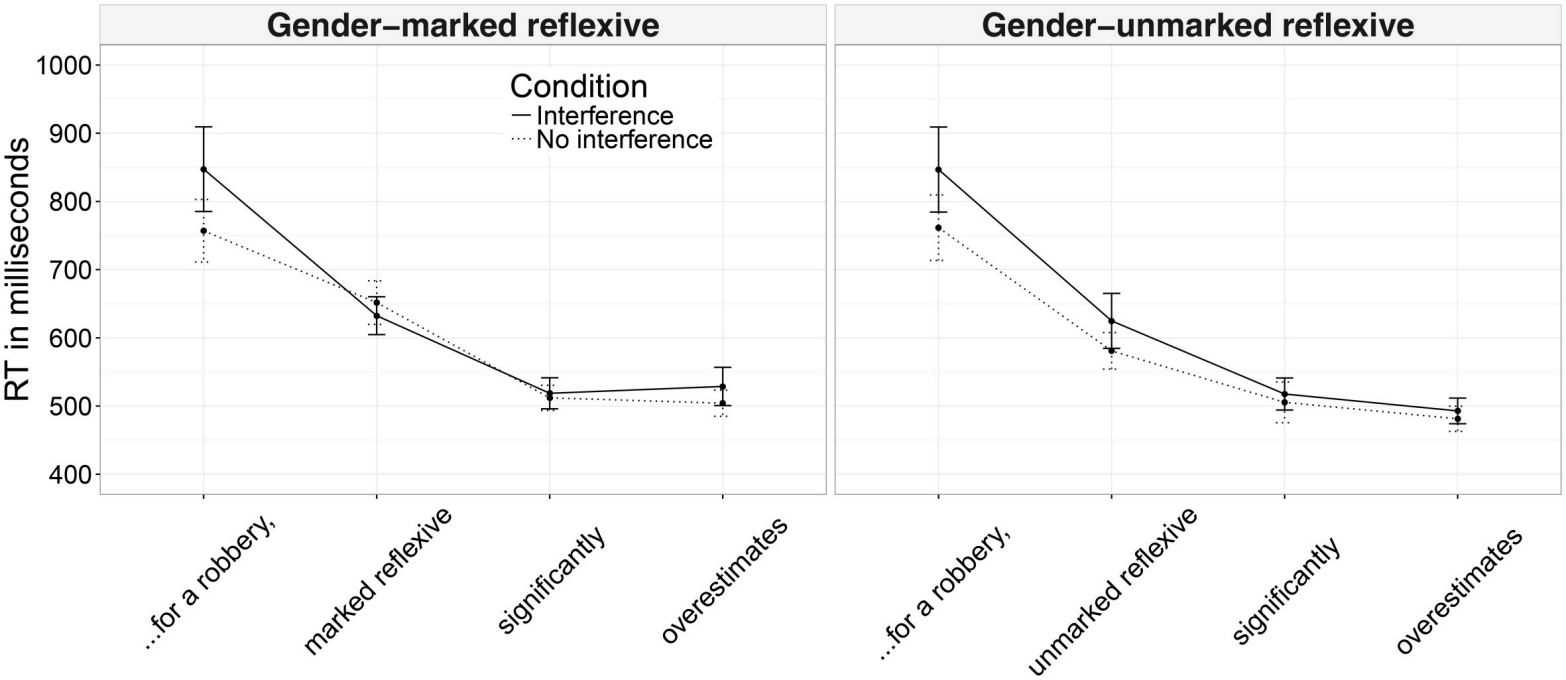
Mean reading times across conditions and their confidence intervals (Experiment 2A).

Mean reading times and their respective confidence intervals for the analyzed regions across conditions are presented in Figure 3.

**Figure 4:**
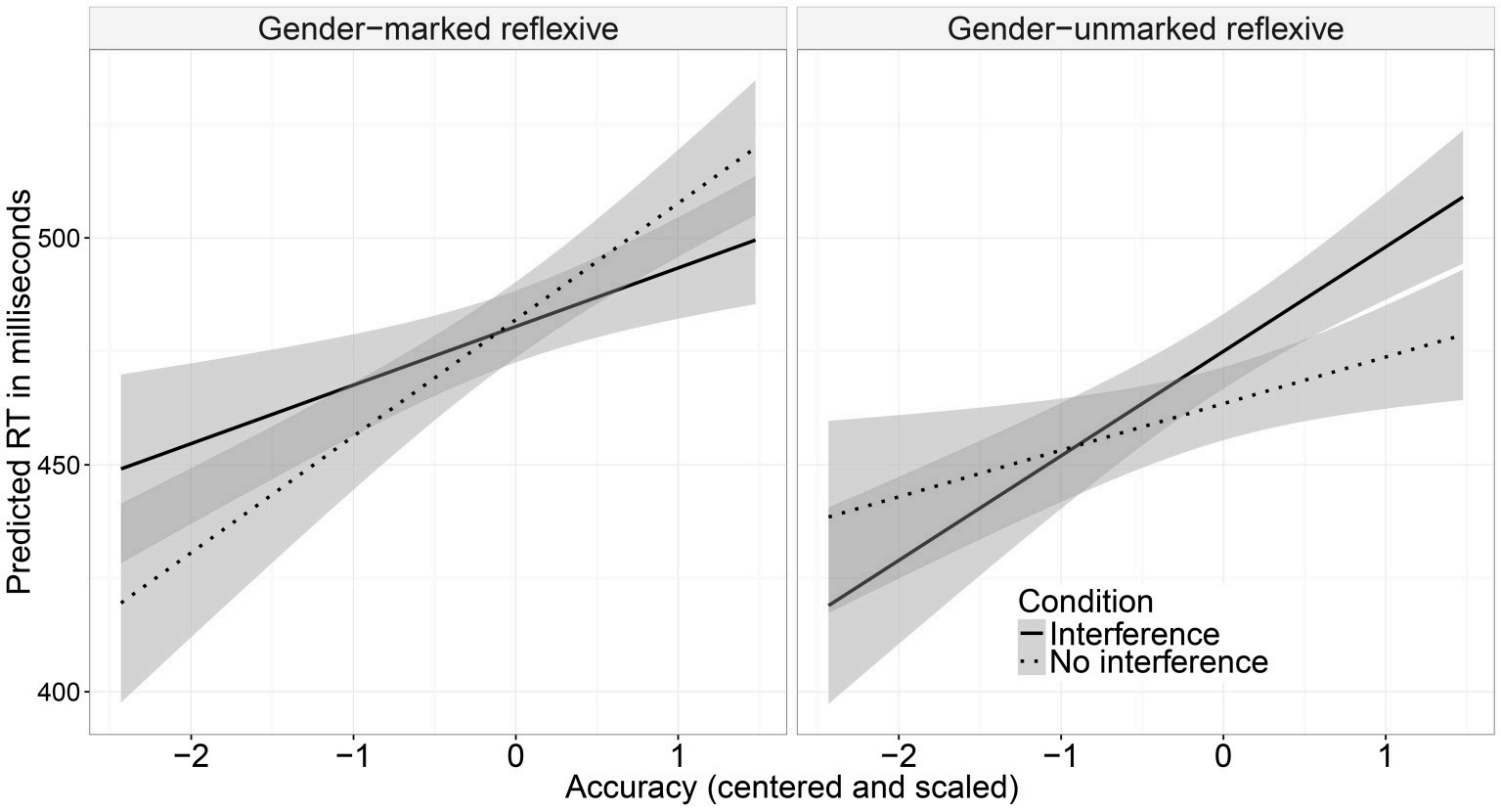
Modeled reading times (and respective standard errors) at the spillover after reflexive (Experiment 2A).

In the region preceding the reflexive, there were main effects of interference (a slowdown in the interference conditions), accuracy (more accurate participants read the region more slowly), and an interaction between these—more accurate participants slowed down even more when the antecedent and the distractor shared the same gender (see Table 4). In the reflexive region, we found a main effect of reflexive type with gender-unmarked reflexives being read faster than gender-marked reflexives, as expected given the respective region lengths. In the region following the reflexive, we found an interaction between reflexive type, interference, and accuracy (see Figure 4). Nested contrasts testing for interference effects within each reflexive type and the interaction between these effects and accuracy did not reach significance. It seems that the interaction was driven by a difference within gender-unmarked reflexives that were read longer by more accurate participants in the interference condition ($\hat{\beta }=-0.013$, *SE* = 0.007, *t* = −1.66 for gender-marked reflexives; $\hat{\beta }=0.013$, *SE* = 0.007, *t* = 1.70 for gender-unmarked reflexives). In the following region (i.e., two words after the reflexive) we again found a main effect of reflexive type (the region was read more slowly in the conditions with gender-marked reflexives) and a main effect of interference (the region was read more slowly when the distractor matched the gender of the antecedent).

**Table 4:** Experiment 2A: Main effects of interference, reflexive type, mean accuracy, and their interactions on log-transformed RTs by regions.

	**Pre-reflexive ***for a robbery*****		**Reflexive ***sebja vs. samu/samogo sebja*****		**Adverb ***significantly*****		**Main verb ***overestimates*****	
	**$\hat{\beta }$(*SE*)**	***t***	**$\hat{\beta }$(*SE*)**	***t***	**$\hat{\beta }$(*SE*)**	***t***	**$\hat{\beta }$(*SE*)**	***t***
Reflexive type	0.003(8)	0.37	0.034(7)	4.77	0.013(7)	1.91	0.024(5)	4.26
Interference	0.025(9)	2.62	0.006(7)	0.89	0.006(6)	1.13	0.012(5)	2.21
Accuracy	0.109(40)	2.70	0.032(25)	1.25	0.031(21)	1.43	0.021(22)	0.93
Int.×Acc.	0.023(10)	2.26	0.011(8)	1.28	0.0002(70)	0.03	0.003(6)	0.54
Int.×Refl.	-0.002(8)	−0.28	−0.007(7)	−0.89	−0.006(6)	−1.07	0.0004(50)	0.08
Int.×Refl.×Acc.	-0.001(8)	−0.17	−0.014(8)	−1.71	−0.013(6)	−2.01	−0.013(7)	-1.89

### 3.5. Discussion

The experiment aimed at determining the type of interference that arises in reflexive processing: the encoding interference account predicts a slowdown in the interference condition independently of reflexive type, while the retrieval interference account predicts an interaction between the reflexive type and interference conditions.

In comprehension questions, similarly to Experiment 1, we observed more errors in the interference (gender match) conditions, irrespective of the reflexive type. This result is in line with with the encoding interference account and might reflect the degraded memory representation of the words that share certain features. An alternative explanation would be that this interference effect is due to some later processing that happens at the moment of answering the comprehension question, rather than due to online processes during reading.

In reading times, we found a main effect of interference at two regions: the word following the verb of the relative clause and the main verb. This is inconsistent with the predictions of the retrieval interference account: as the verbs were not marked for gender, gender could not be used as a retrieval cue, and the amount of retrieval interference should be the same regardless of gender match between the antecedent and the distractor. However, verbs were read more slowly in the conditions where the distractor matched the gender of the antecedent, which could only be explained by encoding interference: as two subjects of their respective clauses that share grammatical gender were written down to memory, their memory representations became less distinguishable, which affected retrieval speed and, consequently, slowed down reading times at the verb regions. Finding consistent evidence for encoding interference in processing subject-verb dependencies is an important result of the present experiment, but it does not necessarily translate to anaphoric dependencies.

The critical interaction that should allow us to disentangle the encoding and retrieval interference accounts in the processing of anaphoric dependencies was found in the region following the reflexive. However, the interaction went into an unexpected direction: we found that gender-unmarked reflexives were read more slowly in the interference condition by accurate participants, while there was no difference in the gender-marked reflexives across conditions. The slowdown in the gender-unmarked reflexives can only be explained by the encoding interference account (and is consistent with the evidence for encoding interference in subject-verb dependencies), but that account predicts a slowdown in the gender-marked reflexives that we do not observe. The retrieval interference account also standardly predicts a slowdown for gender-marked reflexives, although several studies reported a speedup (Sturt, 2003, Experiment 1; Cunnings and Felser, 2013, Experiment 2; Cunnings and Sturt, 2014; Baumann and Yoshida, 2015; Jäger et al., 2015a, Experiment 3). Similarly, in a study on anaphoric noun phrases, Autry and Levine (2014) found that increase in number of potential referents (from two to five) decreased rather then increased reading times at the noun phrase.

Although, our results for the gender-marked reflexives are seemingly in conflict with the predictions of both interference accounts, we propose a *post-hoc* explanation that is consistent with the literature and with the ACT-R model: we suggest that both retrieval and encoding interference affect processing of gender-marked reflexives, and counteract each other. In that case, processing of both the gender-unmarked and the gender-marked reflexives is slowed down in the interference condition, but for the gender-marked reflexives, there is also a speedup in processing due to retrieval interference. Engelmann et al. (2015) have shown that a speedup in the interference condition is actually in line with the retrieval interference as implemented in ACT-R model of sentence processing under certain conditions. Engelmann et al. (2015) demonstrated that if a distractor is particularly activated and matches most of the retrieval cues, it would be misretrieved instead of the antecedent in a large proportion of trials. Due to a race-like scenario, the mean retrieval latencies will be faster in such a configuration (the more items are gaining activation, the sooner on average one of them crosses the activation threshold), which, in turn, would lead to a speedup in mean reading times in the respective condition. Since, we constructed the experimental items such that the distractor is particularly prominent in order to maximize potential retrieval interference effects, it is reasonable to assume that the distractor was highly activated. Two factors contribute to the distractor's prominence: it occupies subject position and stays linearly closer to the reflexive than the antecedent. Earlier we mentioned that being a subject might be one of the retrieval cues (Van Dyke, 2007), and if this is the case, the distractor in our setup matches all but one retrieval cue (being an NP, gender, number, “subjecthood,” but not c-command). Additionally, the meta-analysis (Jäger et al., 2017) shows that distractors that are subjects of their clauses increase the amount of interference. As to recency, it contributes to the base-level activation of an item because ACT-R assumes decay: base-level activation decreases as time since the last retrieval of this item passes. To summarize, there are reasons to believe that in our design, distractors were particularly highly activated, which lead to a speedup due to retrieval interference, and that speedup counteracted the slowdown due to encoding interference in the gender-marked reflexives.

One of the reasons retrieval interference in reflexive dependencies is still a controversial subject is that many studies failed to find interference effects. Among possible reasons could be the insufficient number of participants and resulting low statistical power, or the joint analysis of the data from participants who are accurate in answering comprehension questions and participants who are at chance (see discussion is Section 2.3 and Nicenboim et al., 2015). As could be seen from the results of Experiment 2A, the interference effect is only found in the data from participants who generally answer the comprehension questions above chance. Thus, our results can be seen as an additional evidence for the pattern proposed by Nicenboim et al. (2015): participants who lack the resources to fully parse dependencies and are thus generally poor at answering comprehension questions often rush through the retrieval site and mask the effect that shows up in the data from the more accurate participants.

Another promising account explaining why retrieval interference effects are often not found in English was suggested by Parker and Phillips (2016), who found that illusory negative polarity licensing is modulated by the position of the dependent element with regard to the verb (i.e., *ever* in the *no …ever* dependency). The authors proposed that at the point of processing the verb, the part of sentence that precedes it is consolidated and becomes opaque for retrieval interference. For this reason, they argue, illusory licensing is possible only when both elements precede the verb, and does not occur when the dependent element follows the verb. Parker and Phillips (2016) suggest that the same might be true for reflexive processing. From this point of view, the distractor gets enclosed in the opaque representation that is not able to cause retrieval interference as soon as the main verb in encountered. If the reflexive follows the main verb, it is unable to retrieve the distractor from this representation, and hence no retrieval interference effects are observed at or following the reflexive. Within the ACT-R framework, the position of the reflexive with regard to the main verb is also crucial, albeit for a different reason: the main verb triggers the retrieval of the subject, which is also the reflexive's antecedent. If the reflexive follows the verb and triggers the retrieval of its antecedent, the antecedent is relatively easy to retrieve since it has just received a boost of activation. Consequently, interference from the distractor is less likely to have any measurable effects. This might account for the lack of interference effects in many studies conducted in English, since in English, configurations where the reflexive precedes the main verb are structurally prohibited. There was at least one experiment that aimed at finding interference in a setup where reflexive preceded the verb (in Hindi), but no interference effects were found (Kush and Phillips, 2014). However, in this study the distractor did not bear ergative marking, which might have been one of the retrieval cues for Hindi.

It is possible that in Experiments 1 and 2A the antecedent of the reflexive might have been maintained in focal attention at the point of processing the reflexive, because the antecedent of the reflexive is also a subject that had not yet formed a dependency with the verb. In that case, no retrieval would take place and no retrieval interference is expected. Whether an item in focal attention is predicted to be susceptible to encoding interference, must depend on the model of encoding interference one assumes. No model explicitly posits existence of the focal attention slot, but the model of Oberauer and Kliegl (2006) can be reconciled with it. Since, both the reflexive's antecedent and the distractor are subjects of their respective clauses (and must both be in focal attention at some point during sentence processing), encoding interference might be possible. That account readily accommodates the slowdown in the interference condition for gender-unmarked reflexives, but fails to explain the absence of a slowdown in gender-marked reflexives: if there is no speedup due to retrieval interference, it is unclear why no slowdown due to encoding interference is found in reading times for the gender-marked reflexives. In any case, the focal attention explanation would be ruled out in a setup where the verb precedes the reflexive.

Our third experiment aims at testing Parker and Phillips's (2016) hypothesis that retrieval interference will be blocked if the main verb precedes the reflexive by replicating the second experiment with one important modification—the main verb and the manner adverb that followed the reflexive will now precede it.

## 4. Experiment 2B: russian reflexives, reflexive follows the verb

Experiment 2B seeks to test the hypothesis that the relative order of the reflexive and the main verb might affect the presence of retrieval interference effects. In addition, we expect to replicate the encoding interference effects found in Experiment 2A on the main and relative clause verbs because word order should not affect encoding interference. For example, within the Oberauer and Kliegl (2006) model, both target and distractor have equal chances of losing a feature due to the proposed feature-overwriting mechanism and thus becoming less accessible. Therefore, retrieval of the target item given a feature-sharing distractor should have a longer latency and be more error-prone.

### 4.1. Materials and methods

The experimental materials consisted of the same 32 sets of items as in Experiment 2A. In each sentence, the manner adverb and the main verb were placed between the relative clause and the reflexive. No other changes to the experimental materials were made. An example item is given in (11):

  (11)  a.  **Interference**
               Аферистка_i_, которую **торговка**      нанимает
               Swindler_fem_   whom     **merchant_fem_**  hires
               для ограбления,  серьёзно    переоценивает
               for   robbery,         significantly  overestimates
               **себя_i_/саму себя_i_**            в  способности к
               **self**_**acc**(ø)_/**herself_acc(fem)_**  in ability              to
               обману.
               do trickery.
          b.  **No interference**
               Аферистка_i_, которую **торговец**      нанимает
               Swindler_fem_  whom     **merchant_masc_** hires
               для ограбления, серьёзно   переоценивает
               for   robbery,        significantly overestimates
               **себя_i_/саму себя_i_**           в  способности к
               **self**_**acc**(ø)_/**herself_acc(fem)_** in ability              to
               обману.
               do trickery.
               “The swindler_fem_, whom a merchant_masc/fem_ hires
               for  a   robbery,   significantly   overestimates   her
               own_ø/fem_ trickery skills.”

The same procedure as in Experiment 2A was used, see Section 3.2.

### 4.2. Participants

One-hundred and twelve volunteers who had not participated in the previous experiment took part in the study. All participants were neurologically healthy native Russian speakers and were tested at the Higher School of Economics, Moscow. Their mean age was 26 (range 16–70), 77 participants were female; 15 individuals reported to be left-handed or ambidextrous. The study was approved by the Committee on Interuniversity Surveys and Ethical Assess of Empirical Research of the National Research University Higher School of Economics.

### 4.3. Analysis

The data analysis was analogous to the one of Experiment 2A, see Section 3.3.

### 4.4. Results

#### 4.4.1. Accuracy

The mean accuracy rates by condition and the corresponding standard errors are presented in the Table 5. Participants' mean accuracies in answering antecedent- and distractor-probing questions ranged from 0.27 to 1.00 with a mean of 0.76. Thirty-four out of one-hundred and twelve participants had mean accuracies below chance level (made more than six mistakes).

**Table 5:** Experiment 2B: Mean accuracies and standard errors by condition.

	**Gender-marked**	**Gender-unnmarked**
Interference	0.81 (0.014)	0.76 (0.015)
No interference	0.86 (0.012)	0.85 (0.012)

Statistical analysis revealed a main effect of interference: accuracy was lower in the conditions where the antecedent and the distractor shared the same gender ($\hat{\beta }=-0.27$, *SE* = 0.07, *z* = −4.13, *p* < 0.001). The main effect of reflexive type was also significant: accuracy was lower in conditions with gender-unmarked reflexives ($\hat{\beta }$ = 0.11, *SE* = 0.05, *z* = 2.29, *p* = 0.022). The interaction was not significant.

#### 4.4.2. Reading times

**Figure 5:**
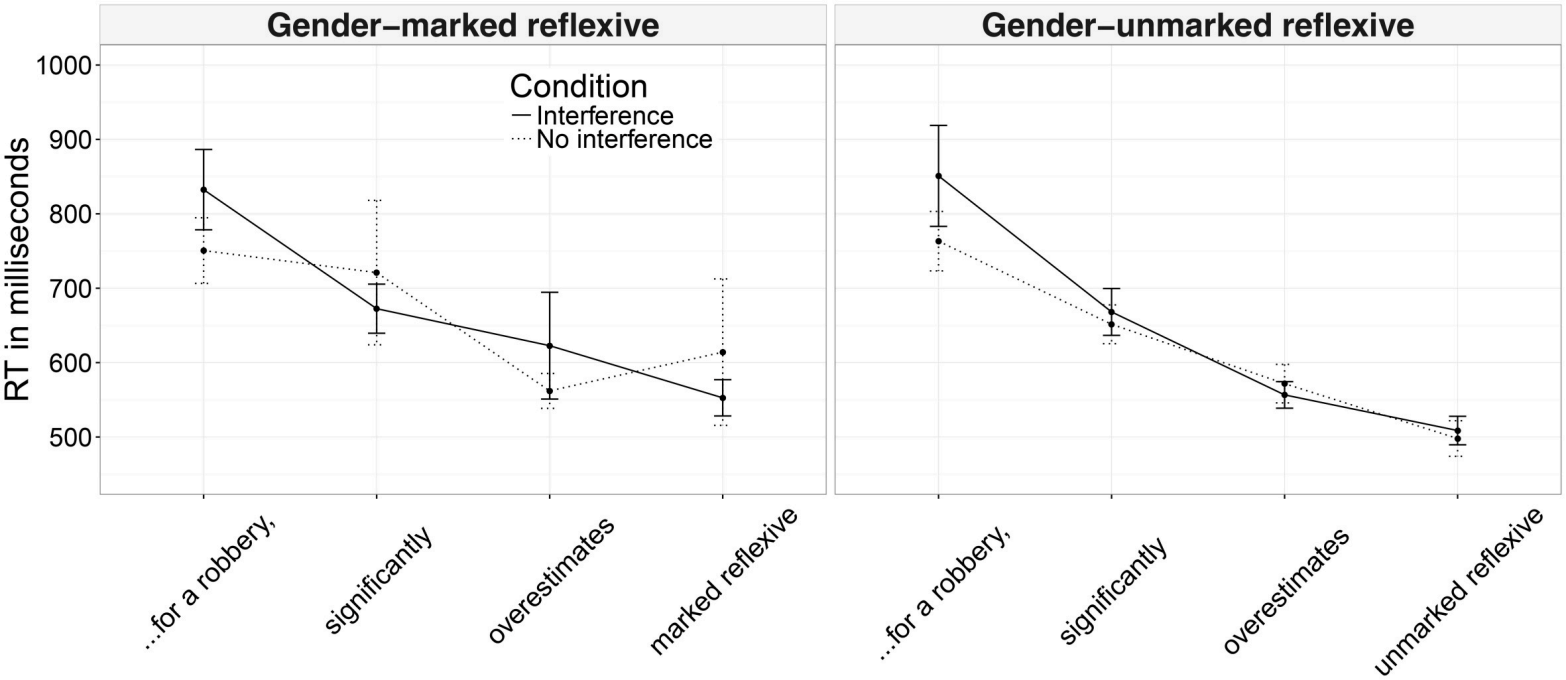
Mean reading times across conditions and their confidence intervals (Experiment 2B).

Mean reading times and their respective confidence intervals for the analyzed regions for each experimental condition are presented in Figure 5.

Main effects of interference and accuracy were found in the region following the verb of the relative clause, : the region was read more slowly by the more accurate participants and in the interference condition (see Table 6). In the two following regions (*significantly overestimates*), a main effect of accuracy was found: accurate participants read these two regions more slowly. In the reflexive region, we found a significant main effect of accuracy (accurate participants read the region more slowly) and an interaction between interference and reflexive type. Nested contrasts testing for interference effects within each reflexive type did not reach significance. It seems that the interaction was driven by the difference between interference and no interference conditions within gender-unmarked reflexives since there was no difference in the gender-marked reflexives ($\hat{\beta }=-$0.006, *SE* = 0.007, *t* = −0.86 for gender-marked reflexives; $\hat{\beta }=0.15$, *SE* = 0.07, *t* = 1.94 for gender-unmarked reflexives).

**Table 6:** Experiment 2B: Main effects of interference, reflexive type, mean accuracy, and their interactions on log-transformed RTs by regions.

	**RC ending for a robbery**		**Adverb significantly**		**Main verb overestimates**		**Reflexive sebja vs. samu/samogo sebja**	
	***$\hat{\beta }$(SE)***	***t***	**$\hat{\beta }$(SE)**	***t***	**$\hat{\beta }$(SE)**	***t***	**$\hat{\beta }$(SE)**	***t***
Reflexive type	−0.006(7)	−0.83	0.007(7)	0.95	0.004(6)	0.70	0.039(6)	5.62
Interference	0.021(8)	2.74	0.002(7)	0.30	0.007(5)	1.37	0.004(5)	0.72
Accuracy	0.138(41)	3.31	0.108(32)	3.35	0.078(29)	2.65	0.064(27)	2.36
Int.×Acc.	0.013(7)	1.77	−0.002(7)	−0.34	0.009(5)	1.69	0.003(5)	0.54
Int.×Refl.	0.0007(70)	0.10	−0.004(6)	−0.72	0.008(5)	1.57	−0.011(5)	−2.08
Int.×Refl.×Acc.	−0.004(7)	−0.60	0.006(6)	0.95	0.005(5)	1.00	0.001(5)	0.37

### 4.5. Discussion

Contrary to what is predicted by both the ACT-R cue-based retrieval model of sentence processing (Lewis and Vasishth, 2005) and Parker and Phillips's (2016) hypothesis (the presence of the verb blocks pre-verbal elements from retrieval interference), in the syntactic configuration where the main verb preceded the reflexive we replicated the main results of Experiment 2A. This means that word order alone cannot explain the absence of interference effects in many studies conducted in English: interference effects are still present in case the verb precedes the reflexive (Nicol and Swinney, 1989; Clifton et al., 1999; Badecker and Straub, 2002, Experiments 5, 6; Sturt, 2003, Experiment 2; Xiang et al., 2009; Clackson et al., 2011; King et al., 2012; Dillon et al., 2013; Parker and Phillips, 2016).

We once again found a correlation between participants' mean accuracy and reading times: in all the analyzed regions, more accurate participants read significantly more slowly. Interestingly, in Experiment 2A, we found this effect only in the pre-critical region and in the spillover after the reflexive. It is unclear why it was not present in other regions, since the accuracies in Experiments 2A and 2B are comparable.

As encoding interference does not depend on the word order, we expected to replicate the encoding interference effects (slower reading times) found in Experiment 2A on the main and relative clause verbs. We found a main effect of interference at the region following the relative clause verb, but not at the main verb. As the region following the main verb was the reflexive, it is impossible to disentangle spillover effects from processing of the reflexive itself. At any rate, the evidence for encoding interference is present in two regions (as compared to three in Experiment 2A): the region following the relative clause verb and the reflexive region.

At the reflexive region, the pattern of reading times is similar to the one observed in Experiment 2A: we again found a slowdown in the interference condition in gender-unmarked, but not gender-marked reflexives, but this time the interaction did not depend on participants' accuracy. The fact that the reading times pattern found in Experiment 2A was again replicated in Experiment 2B is an argument in favor of its systematic nature. However, the *post-hoc* explanation we provided for the effect in Experiment 2A does not fit Experiment 2B equally well: we reasoned that in gender-marked reflexives, the slowdown due to encoding interference is present, but concealed by a speedup caused by retrieval interference. However, the speedup in processing gender-marked reflexives is only predicted by cue-based retrieval as implemented in ACT-R (Lewis and Vasishth, 2005) if the distractor is particularly active. In Experiment 2B, at the point of processing the reflexive the distractor must be less active than the antecedent because of the recent reactivation of the antecedent at the main verb. In such a case retrieval interference account predicts a slowdown at the reflexive region, not a speedup. Therefore, we should observe a slowdown in reading times at gender-marked reflexives when the gender of the distractor matches the gender of the antecedent. Our results contradict this prediction and therefore cannot be reconciled with the retrieval interference account.

If retrieval interference cannot account for the absence of interference effects in gender-marked reflexives, what can? One straightforward option is that gender-marked reflexives differ in some important way from the gender-unmarked reflexives. There is indeed a semantic difference: gender-marked reflexives put emphatic focus on the antecedent. As Lyutikova (1997) puts it, gender-marked reflexives (as opposed to gender-unmarked reflexives that take a purely syntactic function) signal that despite the expectations of a listener, the same person plays two different central roles in the situation (cf. “You did it to yourself”). It means that in our experimental conditions, gender-marked reflexives not only established coreference between the reflexive and the antecedent, but also provided higher-level discourse and/or semantic information, putting the emphatic focus on the antecedent.

Two additional facts may be seen as a *post-hoc* indirect support for the claim that gender-marked reflexives were processed differently. First, in Experiment 2A, there was a main effect of reflexive type two words downstream the reflexive: the word was read longer in conditions with gender-marked reflexives. Second, in Experiment 2B (but not 2A), question response accuracies were higher in conditions with gender-marked reflexives. These results might indicate that processing the emphatic focus on the antecedent took longer than establishing purely syntactic relationship, but the resulting interpretations were more stable, as demonstrated by question response accuracies. However, any *post-hoc* interpretation must remain a speculation until further tests.

Even though gender-marked reflexives might require some additional extra-syntactic processing, at present it is unclear why we did not find encoding interference effects in gender-marked reflexives. Every encoding interference account predicts the same effects regardless of gender marking, and if the slowdown in processing gender-unmarked reflexives is caused by encoding interference, there should be a similar slowdown in processing gender-marked reflexives. We suggest that in sentences with gender-marked reflexives, establishing emphatic focus at the point of retrieving the antecedent is assosiated with greater variance in processing times that conceals the main effect of interference.

An alternative explanation would be that the processing slowdown in marked reflexives might be concealed by a slowdown in the control condition: if on some proportion of trials participants erroneously predicted that the upcoming words should bear the gender marking of the distractor, encountering the gender marking consistent with the target should cause processing delays. No delays of such nature are expected either in the interference condition (since the prediction would always be confirmed), or in the gender-unmarked reflexives (since the prediction could never be disconfirmed). Only in marked reflexives slowdowns might arise in each condition and undermine the comparison between those.

To summarize, in Experiment 2B, we replicated the main results of Experiment 2A: the correlation between reading times and mean accuracies (more accurate participants read more slowly), the encoding interference effects in reading times at the relative clause verb and reflexive, and the unexpected pattern of reading times at reflexive (a slowdown in the interference condition in gender-unmarked, but not gender-marked reflexives). In Experiment 2B, the reading times at reflexive cannot be explained by the retrieval interference account, and the retrieval interference explanation of interference effects in processing reflexives in Russian is therefore ruled out.

## 5. General discussion and conclusions

The main goal of the present paper was to ascertain whether it is retrieval or encoding interference that accounts for the similarity-based interference effects in reflexive processing. The answer to this question would allow us, from the one side, to accept or reject the syntax as an early filter account of sentence processing, and from the other side, to obtain a more general insight into the functioning of working memory in online sentence processing.

In order to disentangle encoding and retrieval interference accounts' predictions, we conducted three experiments: one in German, contrasting reflexive and pronoun processing, and two in Russian, contrasting the processing of gender-marked and gender-unmarked reflexives. In the first experiment, we failed to find any interference effects, presumably due to the difficulty of the experimental materials. In the second experiment, we pitted the predictions of the encoding and the retrieval interference accounts against each other within reflexives: the encoding interference account predicts that both in gender-marked and gender-unmarked reflexives, the interference condition would be processed more slowly. On the contrary, the retrieval interference account predicts that only in the gender-marked reflexives would the difference between the interference and no interference conditions appear, since only in the processing of gender-marked reflexives gender can be used as a retrieval cue. In Experiment 2A, we encountered an unexpected pattern of reading times at the region following the reflexive—a slowdown in the interference condition in the gender-unmarked, but not in the gender-marked reflexives. This reading times pattern was replicated in Experiment 2B, where the order of reflexive and the main verb was reversed (as in English, the reflexive followed the verb). While the results of Experiment 2A might be reconciled with the retrieval interference account under certain conditions, the results of Experiment 2B contradict the predictions of the ACT-R model: when the reflexive is preceded by the verb whose subject is the reflexive's antecedent, retrieval interference effects are expected to lead to a slowdown, not a speedup in mean reading times. Since retrieval interference cannot account for the results of Experiment 2B, and the same pattern of reading times was found in Experiments 2A and 2B, we expect that the underlying cause was the same in both experiments, and therefore the retrieval interference explanation must be rejected. To summarize, we found no retrieval interference effects in the three experiments reported in this paper.

On the contrary, in the two experiments carried out in Russian, we found evidence in favor of encoding, but not retrieval, interference, both in reflexive-antecedent and in subject-verb dependencies. This stands in marked contrast to German, where no encoding interference in the processing of reflexives was found in two high-powered studies (Jäger et al., 2015a) and in the Experiment 1 reported in this paper. It does not seem likely that the existence of encoding interference depends on the language, rather our ability to detect interference effects might depend on the syntactic structure in question and the skill of the readers. As we already noted, the syntactic structure of the sentences used in Experiment 1 was more complicated than that of Experiments 2A and 2B (double vs. single embedding), which might have caused the observed difference across experiments.

At the same time, our results are not fully consistent with the predictions of the encoding interference account: while the slowdown at the reflexive is predicted for all sentences where the distractor matches the gender of the antecedent, we only found it in gender-unmarked, but not in gender-marked reflexives. We suggest that this might have two explanations. The first is that gender-marked reflexives require additional semantic processing: Lyutikova (1997) suggests that in Russian, gender-marked reflexives not only establish referential relationship between the reflexive and its antecedent, but also put emphatic focus on the antecedent. It is possible that additional semantic processing associated with establishing emphatic focus might conceal the encoding interference effect. The second explanation concerns a possible fault in the control condition: if in some proportion of trials participants erroneously expect the gender marking of the distractor on the upcoming words, their predictions can be disconfirmed only in the no interference condition in sentences with gender-marked reflexives. That would lead to delays in reading times, which could in turn undermine the comparison with the interference condition.

Interestingly, consistent evidence for encoding interference was found in the question response accuracies in all three experiments, including the experiment in German. The same pattern of results was also reported for German in Jäger et al. (2015a). Although, this is not explicitly discussed, we assume that both retrieval interference accounts considered in this paper (McElree et al., 2003; Lewis and Vasishth, 2005) predict that in answering comprehension questions, the resulting representation that was built during sentence comprehension is used. Even if later reanalysis was postulated, it would engage the retrieval mechanisms specified in the models. In comprehension questions, the retrieval of verb arguments (required to provide a correct answer) would be initiated at the verb. In all the reported experiments, verbs were gender-unmarked, so gender could not be used as a retrieval cue, and retrieval interference account predicts equal accuracies across all conditions. This contradicts the pattern of observed accuracies. We must either suggest that mechanisms involved in sentence processing and building a faithful representation differ from those that provide access to the resulting representation (as in answering comprehension questions), or interpret comprehension question accuracies as evidence for encoding and against retrieval interference.

Finally, across the three experiments presented in this paper, the correlation between participants' accuracies and reading times seems to be robust: more accurate participants read more slowly. In Experiment 2A, accuracy was crucial for uncovering the critical interaction between interference and reflexive type: the interaction was present only in the more accurate participants' reading times. However, no such relationship was found in Experiment 2B—the critical interaction was not modulated by participants' accuracy. Therefore, we replicated the relationship between reading speed at the retrieval site and comprehension accuracy reported by Nicenboim et al. (2015) only in one of the two experiments. Nevertheless, researchers who investigate long-distance dependencies might benefit from being aware of this relationship and in particular of the fact that reading times from the participants who do not build syntactic dependencies correctly might conceal the effect present in the reading times of the more accurate participants.

To conclude, in two out of three experiments reported in this paper we found a reading times pattern that is inconsistent with the retrieval interference account, but can be explained by encoding interference. Feature-matching distractors influence how coreference between the antecedent and the reflexive is established, and that goes against the strong version of the syntax as an early filer account (Nicol and Swinney, 1989). However, the main claim of the account—that reactivation of the antecedent is restricted by grammatical constraints—still holds true: encoding interference attributes the slowdown in processing the reflexive to feature overwriting and degraded memory representation of the antecedent, not to competition for retrieval between all the nouns.

## Ethics statement

All subjects gave written informed consent in accordance with the Declaration of Helsinki. We did not seek approval by an institutional review board for Experiment 1 because it is not required to conduct a study of the type reported in this manuscript. Experiments 2A and 2B were approved by the Committee on Interuniversity Surveys and Ethical Assess of Empirical Research of the National Research University Higher School of Economics.

## Author contributions

Conception or design of the experiments: AL, LJ, YA, JR, OD; programming of the experiments: AL, JR; data collection: AL, JR; statistical analysis: AL; preparation of the final document: Al, LJ, YA.

### Conflict of interest statement

The authors declare that the research was conducted in the absence of any commercial or financial relationships that could be construed as a potential conflict of interest.

## Footnotes

^1^No difference was found in subject relative clauses, such as 1a.

^2^Hereafter, the illustrations will always refer to the example item, in that case, Example 6.

^3^For the feminine and masculine antecedents: Wilcoxon rank sum test, *W* = 140, *p* = 0.67; for the matching and mismatching distractors: Wilcoxon rank sum test, *W* = 496, *p* = 0.83.
